# Prostate cancer radiotherapy in kidney transplant candidates: a planning framework for defining and optimizing transplant-specific organs at risk

**DOI:** 10.1016/j.ctro.2026.101186

**Published:** 2026-05-15

**Authors:** Anna Gueiderikh, Adrien Panis, Sylvain Klotz, Damien Moreau, Yannis Meraouna, Thomas Bessede, Guillaume Reichert, Charles Dariane, Philippe Giraud

**Affiliations:** a Hôpital Européen Georges-Pompidou, APHP, 20, rue Leblanc, Paris 75015, France; b Hôpital Kremlin Bicêtre, APHP, 78 Rue du Général Leclerc, Le Kremlin-Bicêtre 94270, France

## Abstract

**Purpose:**

Kidney transplant candidates increasingly present with localized or locally advanced prostate cancer. While radiotherapy is frequently indicated, current planning guidelines do not account for the surgical constraints of future kidney transplantation. This study proposes a practical planning framework by defining transplant-specific organs at risk (OARs) and evaluating the dosimetric impact of their systematic optimization during prostate cancer radiotherapy.

**Materials and methods:**

Through multidisciplinary collaboration between radiation oncologists and transplant surgeons, transplantation-critical structures were identified and delineated, including the bladder dome, ureters, and *peri*-vascular iliac fat. A comparative dosimetric analysis was performed across six planning strategies applied to two representative anatomical situations reflecting different bladder volumes: prostate-only Intensity Modulated Radiation Therapy (IMRT) with and without transplant-specific OARs optimization, stereotactic body radiotherapy (SBRT), and prostate plus pelvic nodal IMRT with and without optimization. Dose metrics to transplant-specific and conventional OARs were analyzed.

**Results:**

In prostate-only treatments, active optimization of transplant-specific OARs resulted in a threefold or greater reduction in mean dose to the bladder dome, without compromising target coverage or increasing doses to conventional OARs. Similar dose reductions were observed for ureters and *peri*-vascular iliac external fat. SBRT achieved the lowest absolute doses to all transplant-specific OARs. In contrast, when pelvic lymph nodes were included, optimization failed to meaningfully reduce doses, with mean values exceeding 20 Gy for several transplant-critical structures.

**Conclusion:**

This work establishes a planning framework for integrating kidney transplantation constraints into prostate cancer radiotherapy. Prostate-only radiotherapy allows effective sparing of transplant-critical structures through active optimization, whereas pelvic nodal irradiation inevitably exposes these structures to high doses despite optimization. These findings support transplant-oriented treatment planning and careful selection of pelvic irradiation in kidney transplant candidates.

## Highlights

• First framework integrating kidney transplant constraints in prostate RT.
• Transplant-specific OARs defined: bladder dome, ureters, iliac vessels.
• Dedicated OAR optimization enables substantial dose reduction.
• Prostate-only RT should be considered alongside adapted surgery.
• Supports multidisciplinary planning in kidney transplant candidates.

## Introduction

Prostate cancer (PCa) discovery in kidney transplant candidates is an increasingly frequent situation due to increased transplantation activity and systematic screening of men aged over 50 years (yrs) with a prostate-specific antigen (PSA) testing in the pre-transplantation work-up. The prevalence of PCa in renal transplant candidates ranges from 2% to 5% [1], [2].

Management of PCa in this population generally mirrors that of the broader population and adheres to established clinical guidelines. However, in practice, these typically younger patients more frequently undergo surgery [3], allowing for early undetectable PSA, which offers a shorter delay before transplantation [1]. Radiotherapy (RT) is still the preferred treatment for some kidney transplant candidates —particularly older individuals, those with node-negative T3/T4 tumors, or node-positive disease. Treatment fields are poorly codified: in high-risk patients with node-negative disease, the Association Française d’Urologie (AFU) guidelines advocate against the use of pelvic RT. In node-positive patients, elective nodal irradiation is advised [2]. Although guidelines acknowledge that treatment volumes should be adapted to transplantation projects, no specific RT delineation recommendations are currently available.

Precise target volume definition is particularly critical in kidney transplant candidates, as RT-induced tissue remodeling may directly compromise future transplant feasibility. Indeed, transplantation is usually performed at least three years after RT [1] and prior RT has been shown to impact kidney transplantation surgery. A history of pelvic irradiation is associated with higher operative difficulty, longer operative duration, more frequent occurrence of lymphocele, and more frequent use of uretero-ureteral anastomosis [4], indicating the remodeling of the bladder dome after irradiation. Additionally, ureteral fragility to radiation is demonstrated by the fact that in already transplanted patients, the main complication associated with radiotherapy was obstruction of the terminal ureter with radiation doses ranging from 20 to 45 Gy [5]. Fortunately, a history of pelvic irradiation was not statistically associated with excess graft dysfunction [4], but arteries and soft tissue are also impacted by irradiation. Radiation induces endothelial damage, intimal thickening of large-caliber vessels, and progressive fibrosis, resulting in vascular alterations that may impair the quality of vascular anastomoses during kidney transplantation [6]. Vascular changes to high-caliber arteries were mainly shown after 30 Gy doses [7], but for small-caliber cardiac arteries, a linear relationship was found between dose and cardiac risk with no threshold [8]. No minimum dose to induce vascular atherosclerosis or ureteral changes has been reported. The radiotherapy plan should thus aim to achieve as low as possible doses in those transplant-critical structures.

In light of these considerations, we initiated a collaborative project with the Radiotherapy and Renal Transplant teams of our hospital to define the relevant organs at risk (OARs) for this patient population. Based on real daily cases, a comparative, illustrative dosimetric study was conducted to evaluate the benefits of treatment plan optimization for these OARs and the potential advantages of hypofractionation. Finally, we discuss the optimal approach to treating N+ patients. The objective of this work is to propose a practical planning framework for integrating kidney transplantation constraints into prostate cancer RT, rather than to establish dose–effect relationships.

### Anatomical considerations during surgery

**Fig. 1:**
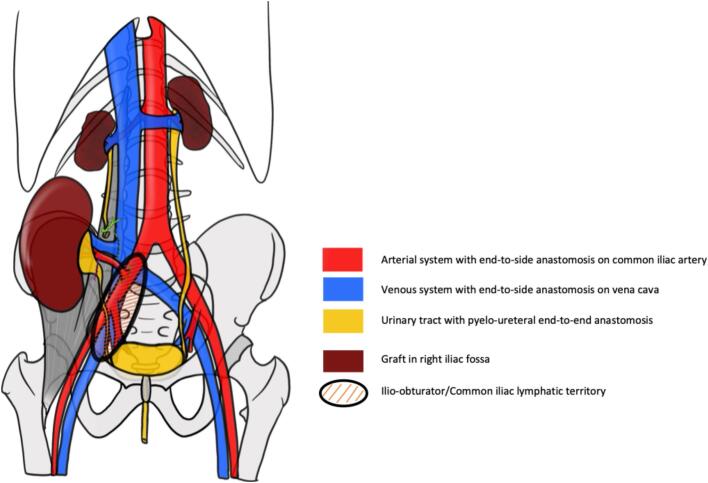
Anatomical scheme of the transplant procedure

Kidney transplantation is the preferred renal replacement therapy for patients with end-stage renal disease [9]. It is most commonly performed through heterotopic implantation of the graft (Fig. 1), whereby the kidney is placed in the retroperitoneal iliac fossa rather than in the native renal position. The procedure requires dissection of the iliac vessels to isolate the artery and vein, vascular anastomoses between the graft renal vessels and the recipient’s iliac vessels, as well as ureteral reimplantation into the bladder, which is often at the bladder dome. These surgical steps involve extensive dissection within the iliac fossa and direct manipulation of vascular and urinary structures which may be exposed to pelvic irradiation before kidney transplantation. Based on our institutional experience, the final positioning of the graft within the iliac fossa itself is not impaired by prior radiotherapy.

The most widely used transplantation technique, initially described by René Küss in 1951 [10], relies on vascular anastomoses to the external iliac vessels. While early procedures involved end-to-end anastomosis to the internal iliac artery, current practice uses end-to-side anastomoses to both the external iliac artery and vein. Following vascular reconstruction, urinary continuity is typically restored through an anti-reflux ureteroneocystostomy, most commonly using the Lich–Gregoir technique [11]. In order to perform ureteral implantation, particular attention must be given to bladder integrity, compliancy and alterations related to chronic kidney disease or prior pelvic irradiation. Indeed, they may increase the risk of complications such as ureterovesical stenosis or vesicoureteral reflux, potentially affecting graft function.

An alternative technique developed at Necker Hospital consists of implanting the graft in the iliac fossa, with vascular anastomoses carried out more proximally, at the aortic bifurcation and the origin of the inferior vena cava [12].

In selected cases, urinary drainage is achieved through an end-to-end uretero-ureterostomy using the recipient’s native ureter, thereby preserving the pelvic ureter (Fig. 1). This strategy may reduce certain urinary complications and highlights the importance of maintaining ureteral integrity during pelvic treatments. Implantation side selection depends on recipient-specific factors, including iliac artery calcification, prior pelvic surgery or irradiation, and vessel depth. When both sides are suitable, the right iliac fossa is generally preferred because of easier surgical access. Graft survival averages approximately 14 years for deceased-donor transplants and up to 20 years for living-donor transplants [13], therefore retransplantation represents a realistic scenario for many patients. Subsequent grafts are generally implanted on the contralateral iliac vessels, highlighting the importance of preserving both iliac vascular axes.

These anatomical and surgical considerations inform radiotherapy planning in kidney transplant candidates and highlight the need to minimize radiation exposure to structures critical to current or future transplantation.

## Material and methods

OARs were defined based on anatomical considerations relevant to transplantation surgery, through structured collaborative agreement between the radiotherapy and surgical transplantation teams.

**Fig. 2:**
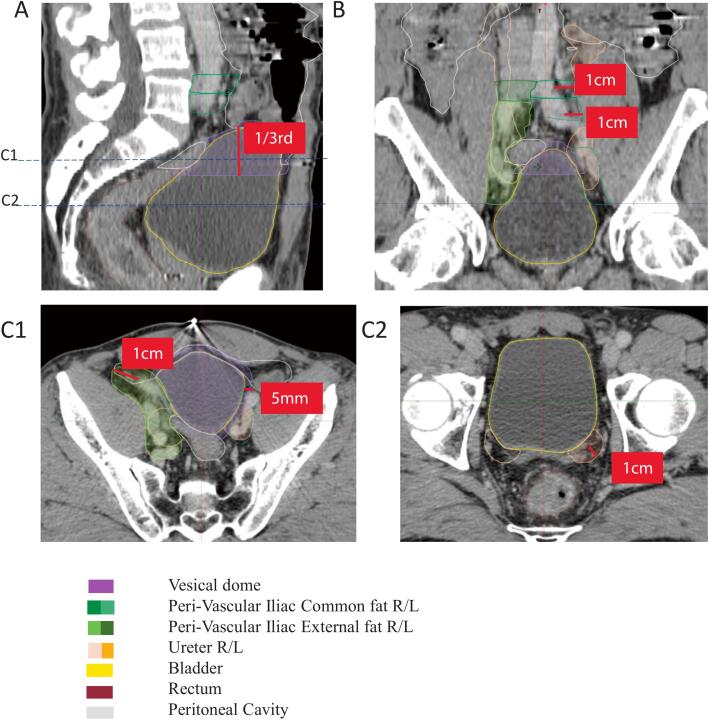
Obtained OARs volumes

Proposed OARs are described in Table 1 and shown in Fig. 2. All OARs have been delimited bilaterally as a second contralateral transplantation can occur over time.

**Table 1:** Definition of the proposed OARs

	Upper limit	Lower limit	Lateral and Antero-posterior limits
Peri-vascular Iliac common fat	Aortic bifurcation	Iliac common bifurcation	Retroperitoneal fat, extending at least 1cm around the iliac common vessels excluding peritoneal cavity, bladder, muscles and bone
Peri-vascular iliac external fat	Iliac common bifurcation	Upper limit of acetabulum	At least 1cm around the iliac external vessels, including all the pre-vascular fat anteriorly, excluding peritoneal cavity, bladder, muscles and bone. Thus volume is delineated independently from the Iliac internal vessels
Bladder dome	Top of the bladder with a 5mm extension	Upper 1/3 of bladder	Whole bladder with 5mm margins laterally and anteriorly
Ureters	Renal pyelon	Uretero-vesical junction	Fat extending 1cm around the ureter, excluding muscles, peritoneal cavity and bladder

**Peri-vascular space** was divided in **Iliac common** and **Iliac external** structures to report properly the dose delivered. It was not contoured as the lymphatic target in a classical nodal irradiation but aimed to encompass the fat and the conjunctive tissue around the vessels, which can be implied in the fibrosis process. A 1 cm margin around the vessels was chosen. Iliac internal vessels were not considered for the volume definition; they could be partly or totally included on some CT slices. The volumes were cropped for bladder, gastrointestinal tract, muscles and bone.

**Bladder dome** was defined as the cranial one-third of the bladder along the superior–inferior axis, with a 5mm extension in the surrounding conjunctive tissue.

**Ureters** were delimited from their bladder implantation up to the kidney and 1cm extent was added. The volumes were cropped for bladder, gastrointestinal tract, muscles and bone.

Usual prostatic organs at risk were also delineated. Sigmoid bowel was included in the peritoneal cavity structure but was also delineated separately for the dosimetric analysis of this project.

Six dosimetric scenarios were then evaluated to assess the reported doses to these OARs. Four scenarios were applied to prostate-only treatment plans. The CTV 76 Gy encompassed the prostate and the proximal one-third of the seminal vesicles, while CTV 46 Gy included the entire seminal vesicles, in accordance with current RECORAD guidelines for high-risk tumors [14].

The first scenario consisted of an IMRT plan without optimization on the proposed OARs (scenario 1). The second scenario corresponded to an IMRT plan with optimization on these OARs (scenario 2). The third scenario consisted of a prostate stereotactic plan delivered using a CyberKnife® system, without optimization on the proposed OARs (scenario 3). The fourth scenario was a prostate stereotactic plan using the same device but with optimization on the proposed OAR (scenario 4).

The fifth and sixth scenarios were applied to prostate and pelvic lymph node radiotherapy. In these scenarios, CTV 76 Gy encompassed the prostate and the proximal one-third of the seminal vesicles, while CTV 46 Gy included the entire seminal vesicles and the pelvic lymph node regions. The fifth scenario consisted of an IMRT plan without optimization on the proposed OARs (scenario 5), and the sixth scenario corresponded to an IMRT plan with optimization on these OARs (scenario 6). All scenarios were applied to two representative anatomical situations derived from clinical CT datasets, selected to reflect different bladder volumes. The first corresponded to a bladder volume of 800 cc, and the second to 250 cc. Each CT dataset was derived from a different patient and reflected the optimal bladder filling achieved in that individual. Bladder filling was obtained using a standardized protocol, with patients instructed to drink 500 cc of water 30 minutes prior to CT acquisition.

ICRU 83 – guided coverage of the PTV and maximum dose tolerated to the usual OARs complying to the RECORAD guidelines [14] were used. Dose constraints used for plan building are detailed in Supplementary Tables 1 and 2. Further optimization was performed to lower to maximum the dose received by the new transplan-specific OARs, on a case-by case basis.

This planning study used anonymized imaging datasets and did not require formal ethics approval according to institutional policy.

## Results

### Dosimetric study

**Fig. 3:**
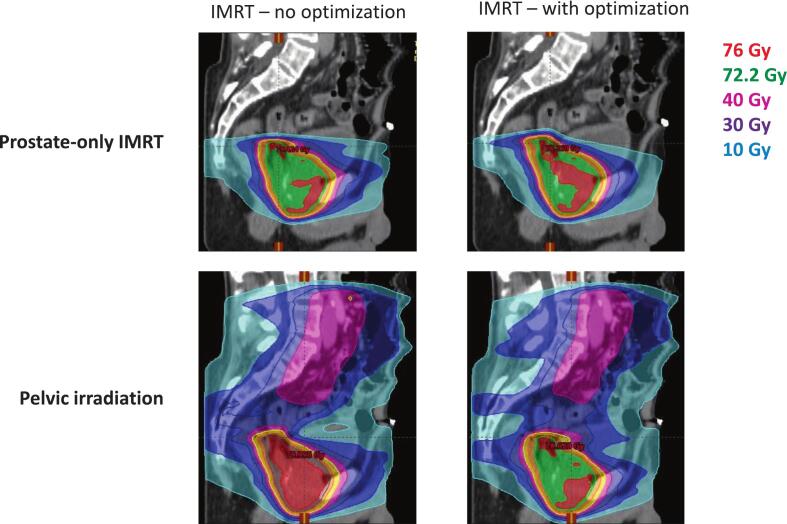
Obtained dose-distribution with and without optimization on transplant-specific OARs in the low-bladder volume situation

We compared two IMRT plans: with or without dose optimization on the proposed OARs. We showed that for both anatomical situations, optimization on the proposed OARs enabled a threefold or more reduction of the dose to the bladder dome. Mean dose decreased from 11.6 to 4.4 Gy in the low-volume-bladder-scenario and from 1.1 to 0.4 Gy in the high-volume-bladder-scenario (Table 2 and Fig. 3). Dose reduction to the peri-vascular fat (mean dose reduction from 1.8 Gy to 0.5 Gy in the high-volume-bladder scenario) was also achieved (Supplementary Table 3). Dose reduction to the ureters was more limited, particularly in low-bladder-volume scenario, reflecting their proximity to the prostate target. Vascular iliac common structures received less than 1 Gy in all three prostate plans. The dose reduction after optimization was not at the detriment of common OARs such as rectum and bladder. Reported doses were similar for left and right structures. (Complete dose report can be found in Supplementary Table 3).

**Table 2:** Mean dose (Gy) to the bladder dome across planning strategies

**Planning strategy**	**High-bladder-volume situation**	**Low-bladder-volume situation**
**Prostate-only IMRT**		
IMRT – no optimization	1.1 [0.5–3.4]	11.6 [1.7–60.6]
IMRT – with optimization	**0.4** [0.2–0.9]	**4.4** [1.4–40.8]

**SBRT**		
SBRT – no optimization	0.1 [0.0–0.4]	7.0 [1.4–12.2]
SBRT – with optimization	**0.1** [0.0–0.3]	**3.8** [0.7–7.2]

**Pelvic irradiation**		
Pelvis IMRT – no optimization	20.8 [2.7–46.8]	32.2 [11.3–68.2]
Pelvis IMRT – with optimization	**19.8** [2.2–46.7]	**17.8** [8.7–54.8]

Data are reported as mean dose [range of the DVH curve], in Gy. Bold values indicate mean doses obtained with optimization.

Those doses can be compared to doses achieved with a not-optimized SBRT plan and to the doses achieved with a plan that includes the pelvic volume (Table 2 and Supplementary Tables 3 and 4). The SBRT plan showed the lowest mean dose to the bladder dome in the high-volume situation (0.1 Gy) but the optimized IMRT plan could outperform it in the low-bladder volume situation (mean dose of 4.4 Gy in the low-bladder-volume situation for the IMRT plan). The SBRT plan offered however the lowest maximal doses to all transplant-related OARs (Supplementary Table 3). Further optimization of the SBRT plan on the transplant-specific OARs enabled further lowering the maximal dose to those structutres in the low-bladder volume situation (Table 2 and Supplementary Table 3). Doses to the bladder dome are similar with the IMRT and the SBRT technique in this anatomical situation.

Pelvic irradiation exposed the bladder dome to mean doses ranging from 17.8 Gy to 19.8 Gy despite the use of optimization (Table 2).

### Transplant-oriented planning workflow

**Fig. 4:**
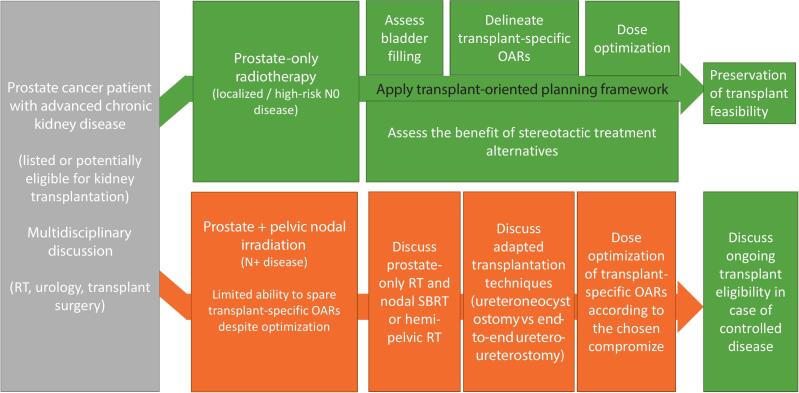
Transplant-oriented radiotherapy planning workflow for prostate cancer in kidney transplant candidates

Taking into account all those data, we propose the transplant-oriented planning workflow as described in Fig. 4.

This figure illustrates how integration of transplant-specific organs at risk into radiotherapy planning may improve RT in kidney transplant candidates. While prostate-only radiotherapy allows effective dose optimization and preservation of transplant feasibility, pelvic nodal irradiation implies unavoidable exposure of transplant-critical structures.

## Discussion

This study represents the first systematic effort to define transplantation-specific OARs for prostate cancer RT planning in kidney transplant candidates. This work should be considered a planning framework rather than a dose–effect or outcome-driven study. By establishing practical delineation guidelines and assessing their dosimetric impact, we provide a framework to optimally preserve future transplant options. Given the absence of established dose thresholds for radiation-induced vascular, ureteral or bladder dome injury in the transplant setting, dose minimization to transplant-critical structures should be considered a valid planning objective per se.

We highlight the contouring of the bladder dome, ureters and peri-vascular iliac external fat as of particular interest to the surgeon. Our dosimetric analysis demonstrates that prostate-only treatment with plan optimization can achieve sparing of those transplant-critical structures. The bladder dome and ureters benefited most from optimization, with mean doses to the bladder dome decreasing by 60-70% without compromising target coverage or increasing doses to standard OARs. Plan optimization must be combined with appropriate bladder filling protocols, as our results demonstrate that bladder repletion is critical for dose reduction. Stereotactic body radiotherapy achieved the lowest maximal doses to all proposed OARs however, bladder filling remained essential to minimize the dose to the bladder dome. Stereotaxic irradiation may offer an advantage in some patients, but its benefit may be modest, especially in those who can fill their bladder correctly. However, we think that one should turn to SBRT use only if the IMRT plan is unacceptable, as the tumors treated with RT are advanced in this population and SBRT is not used in those indications in everyday practice.

From a surgical perspective, adaptation of the implantation technique may also be considered in previously irradiated patients. The Necker approach, which involves higher vascular anastomoses on the common iliac vessels, may theoretically reduce exposure of critical vascular axes, as these structures receive lower doses than the external iliac vessels in standard pelvic radiotherapy fields. In this context, our surgical team may favor uretero-ureteral or pyelo-ureteral anastomosis to avoid additional pelvic dissection toward the bladder dome, although the risk of ureteral stenosis must be acknowledged.

For teams less familiar with this implantation technique, our dosimetric data demonstrate that careful radiotherapy optimization combined with adequate bladder filling can effectively spare the external iliac vessels, with reported doses ranging from 0.5 to 2 Gy in optimized prostate-only plans. Radiotherapy optimization therefore plays an important role not only in treatment delivery but also in facilitating multidisciplinary discussion regarding the most appropriate implantation strategy.

Pelvic nodal irradiation represents a fundamentally different planning scenario rather than a simple extension of prostate-only radiotherapy. Our dosimetric analysis shows that, when pelvic lymph nodes are included in the treatment volume, optimization fails to meaningfully reduce dose exposure to transplant-specific organs at risk, with mean doses consistently exceeding 17 Gy. In particular, peri-vascular iliac external fat received mean doses ranging from 27 to 47 Gy, ureters received mean doses between 11 and 36 Gy and the bladder dome received doses between 17 and 20 Gy. While direct dose–effect relationships for surgical complications in this setting remain undefined, such dose levels are likely to increase surgical complexity through radiation-induced fibrotic and vascular changes. As the bladder dome and ureter receive both increased RT doses, neither transplantation technique can be considered as spared in case of complete pelvic nodal irradiation.

These findings should be interpreted in the context of evolving evidence regarding the oncological benefit of pelvic nodal irradiation. The RTOG 0924 trial, presented at ASTRO 2025, reported no improvement in metastasis-free survival and only a modest benefit in biochemical control for pelvic radiotherapy in high-risk prostate cancer patients [15]. In kidney transplant candidates, these results support no use of pelvic RT in node-negative patients and a cautious and highly selective use of pelvic irradiation in node-positive patients, as any potential oncological benefit must be carefully balanced against the impact on future transplant feasibility. Surgical alternatives, such as extended lymphadenectomy, are also constrained in this population, as nodal dissection may compromise subsequent graft implantation. Accordingly, AFU guidelines recommend nodal dissection primarily in patients with proven nodal involvement [2], reflecting a similar balance between oncological control and preservation of transplant options. Although the PEACE-V–STORM trial demonstrated improved oncological outcomes with whole pelvic radiotherapy compared with metastasis-directed therapy in the general oligometastatic population [16], the unique anatomical and surgical constraints of transplant candidates may justify individualized treatment strategies, even at the cost of potentially reduced oncological efficacy [17]. In this context, optimization of the proposed transplant-specific OARs should be applied as described. Treatment planning should take into account the anticipated transplant trajectory, including the potential need to adapt the transplantation technique and ureteral implantation site. Depending on nodal location, prioritization of dose sparing may differ between the bladder dome and the ureter.

Our study primarily focused on IMRT plan optimization, as this remains the most commonly used radiotherapy modality in kidney transplant candidates, who often present with or high or very high-risk disease, including node-negative T3/T4 tumors or node-positive disease. A conventionally fractionated IMRT treatment schedule was therefore considered the reference treatment. A hypofractionated IMRT treatment plan would benefit to a similar extent from transplant-specific OAR optimization. Indeed, to date, these patients are predominantly treated with IMRT, although SBRT is gaining increasing interest in more advanced disease settings, notably following the PACE trials. In particular, the PACE-B trial demonstrated the non-inferiority of SBRT in intermediate-risk prostate cancer [18], while PACE-C reported no excess toxicity in high-risk node-negative disease [19], with efficacy outcomes still awaited. In this context, SBRT was included in our analysis as an exploratory comparison to estimate the magnitude of dose reduction achievable with highly conformal techniques. SBRT may gain broader indications in the coming years, which could further expand the role of radiotherapy in this population. Our results show that optimization on transplant – specific OARs remains of interest in some patients treated with SBRT. Adaptive radiotherapy approaches, such as MR-guided radiotherapy, may further improve treatment optimization by accounting for daily anatomical variations.

This study has several important limitations. The dosimetric analysis was performed on only two anatomical situations, limiting generalizability, though these cases represent realistic clinical situations with different bladder volumes. The limited number of situations does not undermine the validity of the proposed OARs definitions, which are anatomy-driven and reproducible. This study establishes technical feasibility but cannot assess the impact of our proposed dose constraints on actual transplant outcomes, surgical complications, or oncological control. For publication clarity, contours were created on contrast-enhanced CT scans, though in clinical practice these patients often undergo non-contrast imaging due to renal insufficiency. Our OARs definitions used easily identifiable structures to account for this limitation. Multi-center prospective studies tracking surgical outcomes, graft function, and oncological control are essential to establish evidence-based dose constraints. The impact of hypofractionated regimens on transplant outcomes also requires investigation. The value of this work lies in systematic organ-at-risk definition and planning principles that can be readily implemented in daily practice, independently of clinical outcome data.

This work establishes the first systematic framework for defining and optimizing transplantation-specific organs at risk in prostate cancer radiotherapy planning. By systematically integrating kidney transplantation constraints into treatment planning, it enables proactive preservation of future transplant feasibility, particularly in prostate-only radiotherapy scenarios. In contrast, pelvic nodal irradiation represents a paradigm shift rather than a technical optimization problem, with unavoidable exposure of transplant-critical structures. These planning principles may be relevant not only for patients currently listed for transplantation, but also for individuals with advanced chronic kidney disease whose transplant eligibility may evolve over time. These principles may be readily implemented in daily practice and support early multidisciplinary discussion in kidney transplant candidates requiring prostate radiotherapy.

## CRediT authorship contribution statement

**Anna Gueiderikh:** Writing – original draft, Methodology, Investigation, Conceptualization. **Adrien Panis:** Writing – original draft, Conceptualization. **Sylvain Klotz:** Investigation, Formal analysis. **Damien Moreau:** Investigation, Formal analysis. **Yannis Meraouna:** Writing – review & editing, Conceptualization. **Thomas Bessede:** Writing – original draft. **Guillaume Reichert:** Conceptualization. **Charles Dariane:** Writing – review & editing, Conceptualization. **Philippe Giraud:** Writing – review & editing, Supervision, Project administration, Conceptualization.

## Declaration of competing interest

The authors declare that they have no known competing financial interests or personal relationships that could have appeared to influence the work reported in this paper.
