# Supplementary material for: Prostate cancer radiotherapy in kidney transplant candidates: a planning framework for defining and optimizing transplant-specific organs at risk
Source: Clin Transl Radiat Oncol. 2026 May 15;59:101186. doi: 10.1016/j.ctro.2026.101186 (PMC13214259; doi:10.1016/j.ctro.2026.101186)
Supplement: Supplementary Data 1 [file mmc1.docx]

**Supplementary Table 1 : Dose constraints used to build the non-optimized IMRT plan.**

|  | **Structure** | **Objective** | **Variation** |
| --- | --- | --- | --- |
| **Target volume objectives (Priority 1)** | | | |
|  | **PTV 76** | D98% > 72.2 Gy |  |
|  | **PTV 76** | D2% < 79.8 Gy |  |
|  | **PTV 70** | D98% > 66.5 Gy |  |
|  | **PTV 46** | D98% > 43.7 Gy |  |
| **Standard OAR constraints (Priority 2)** | | | |
|  | **Bladder wall** | Dmax < 78 Gy | < 79 Gy |
|  | **Bladder wall** | D2% < 78 Gy |  |
|  | **Bladder wall** | V70Gy < 25% |  |
|  | **Bladder wall** | V60Gy < 50% |  |
|  | **Rectal wall** | Dmax < 74 Gy | < 75 Gy |
|  | **Rectal wall** | D2% < 74 Gy |  |
|  | **Rectal wall** | V70Gy < 20% | < 25% |
|  | **Rectal wall** | V60Gy < 35% | < 50% |
|  | **Rectal wall** | V50Gy < 50% |  |
|  | **Rectal wall** | V47Gy < 53% |  |
|  | **Small bowel** | V15Gy < 830 cm³ |  |
|  | **Small bowel** | V30Gy < 500 cm³ |  |
|  | **Small bowel** | V45Gy < 150 cm³ |  |
| **Secondary OAR constraints (Priority 3)** | | | |
|  | **Sacrum** | D2% < 50 Gy | < 55 Gy |
|  | **Left femoral head** | D2% < 50 Gy | < 55 Gy |
|  | **Right femoral head** | D2% < 50 Gy | < 55 Gy |
| **Additional constraints (Priority 4 / Reference)** | | | |
|  | **External genitalia** | D20% < 30 Gy |  |
|  | **Penile bulb** | Dmax < 76 Gy | < 80 Gy |

**Supplementary Table 2 : Dose constraints used to perform the non-optimized SBRT plan.**

|  | **Structure** | **Objective** | **Variation** |
| --- | --- | --- | --- |
| **Target volume objectives (Priority 1)** | | | |
|  | **PTV High** | V36.25 Gy ≥ 98% | > 95% |
|  | **Prostate** | V36.25 Gy = 100% |  |
| **Plan quality objective (Priority 2)** | | | |
|  | **PTV High** | nCI ≤ 1.01 |  |
| **Critical OAR constraints (Priority 2)** | | | |
|  | **Cauda equina** | D0.04cc < 25.3 Gy |  |
|  | **Cauda equina** | V30Gy < 3 cc |  |
|  | **Lumbosacral plexus** | D0.04cc < 32 Gy |  |
|  | **Lumbosacral plexus** | V30Gy < 3 cc |  |
| **Standard OAR constraints (Priority 3)** | | | |
|  | **Bladder wall** | D0.04cc < 38 Gy |  |
|  | **Bladder wall** | V18Gy < 15 cc |  |
|  | **Rectal wall** | D0.04cc < 38 Gy |  |
|  | **Rectal wall** | V25Gy < 20 cc |  |
|  | **Small bowel** | D0.04cc < 29 Gy |  |
|  | **Small bowel** | V19.5Gy < 5 cc |  |
|  | **Skin** | D0.04cc < 32 Gy |  |
|  | **Skin** | V30Gy < 10 cc |  |
| **Secondary OAR constraints (Priority 4)** | | | |
|  | **Right femoral head** | V30Gy < 10 cc |  |
|  | **Left femoral head** | V30Gy < 10 cc |  |

**Supplementary tables 3a and 3b: Obtained Doses to OARs in the Four Scenarios - Prostate Only Treatment**

**3a) Anatomical situation with High Volume Bladder (800 cc)**

Data are reported as mean dose [range of the DVH curve], in Gy. Bold values indicate doses obtained with optimization.

| **Structure** | **Scenario 1 (IMRT, no optimization)** | **Scenario 2 (IMRT, with optimization)** | **Scenario 3 (SBRT, no optimization)** | **Scenario 4 (SBRT, with optimization)** |
| --- | --- | --- | --- | --- |
| **Peritoneal cavity** | 0.2 [0.0; 0.9] | **0.1 [0.0; 0.8]** | 0.0 [0.0; 0.2] | **0.0 [0.0; 0.2]** |
| **Sigmoid** | 0.8 [0.2; 2.6] | **0.3 [0.1; 0.8]** | 0.0 [0.0; 0.2] | **0.0 [0.0; 0.2]** |
| **Rectum** | 32.0 [2.4; 74.0] | **23.0 [0.8; 76.0]** | 5.5 [0.2; 38.1] | **5.6 [0.2; 38.1]** |
| **Bladder** | 22.4 [0.6; 77.8] | **13.0 [0.2; 78.8]** | 4.1 [0.0; 38.4] | **4.1 [0.0; 38.4]** |
| **Peri-vascular iliac common fat (R)** | 0.4 [0.3; 0.5] | **0.2 [0.1; 0.2]** | 0.0 [0.0; 0.0] | **0.0 [0.0; 0.0]** |
| **Peri-vascular iliac common fat (L)** | 0.4 [0.3; 0.4] | **0.2 [0.1; 0.2]** | 0.0 [0.0; 0.0] | **0.0 [0.0; 0.0]** |
| **Peri-vascular iliac external fat (R)** | 1.9 [0.4; 10.3] | **0.6 [0.2; 1.6]** | 0.6 [0.0; 7.6] | **0.6 [0.0; 7.6]** |
| **Peri-vascular iliac external fat (L)** | 1.7 [0.4; 10.0] | **0.5 [0.2; 1.5]** | 0.5 [0.0; 4.7] | **0.5 [0.0; 4.7]** |
| **Bladder dome** | 1.2 [0.5; 3.4] | **0.5 [0.2; 0.9]** | 0.1 [0.0; 0.4] | **0.1 [0.0; 0.4]** |
| **Ureter (R)** | 0.4 [0.0; 69.5] | **0.4 [0.0; 4.4]** | 0.3 [0.0; 5.0] | **0.1 [0.0; 1.6]** |
| **Ureter (L)** | 0.4 [0.0; 76.6] | **0.4 [0.0; 6.8]** | 0.4 [0.0; 8.3] | **0.1 [0.0; 3.1]** |

**3b) Anatomical situation with Low Volume Bladder (250 cc)**

Data are reported as mean dose [range of the DVH curve], in Gy. Bold values indicate doses obtained with optimization.

| **Structure** | **Scenario 1 (IMRT, no optimization)** | **Scenario 2 (IMRT, with optimization)** | **Scenario 3 (SBRT, no optimization)** | **Scenario 4 (SBRT, with optimization)** |
| --- | --- | --- | --- | --- |
| **Peritoneal cavity** | 0.4 [0.0; 57.9] | **0.4 [0.0; 60.6]** | 0.5 [0.0; 10.1] | **0.2 [0.0; 8.6]** |
| **Sigmoid** | 5.4 [1.3; 57.9] | **5.2 [1.3; 60.8]** | 2.0 [0.3; 9.9] | **1.3 [0.2; 7.0]** |
| **Rectum** | 37.4 [3.4; 75.9] | **38.1 [3.0; 76.8]** | 8.2 [0.4; 38.3] | **8.4 [0.4; 38.4]** |
| **Bladder** | 34.9 [2.0; 77.2] | **27.1 [1.5; 77.0]** | 14.5 [1.9; 37.9] | **11.9 [1.1; 38.4]** |
| **Peri-vascular iliac common fat (R)** | 0.4 [0.2; 0.9] | **0.4 [0.2; 0.9]** | 0.1 [0.0; 0.2] | **0.0 [0.0; 0.1]** |
| **Peri-vascular iliac common fat (L)** | 0.4 [0.2; 0.9] | **0.4 [0.2; 0.9]** | 0.1 [0.0; 0.3] | **0.0 [0.0; 0.1]** |
| **Peri-vascular iliac external fat (R)** | 2.4 [0.7; 18.9] | **1.8 [0.6; 13.2]** | 3.4 [0.2; 9.9] | **2.0 [0.1; 7.6]** |
| **Peri-vascular iliac external fat (L)** | 2.8 [0.6; 18.6] | **2.0 [0.6; 11.7]** | 3.9 [0.2; 10.3] | **2.4 [0.1; 9.7]** |
| **Bladder dome** | 11.6 [1.7; 60.6] | **4.4 [1.4; 40.8]** | 7.0 [1.4; 12.2] | **3.8 [0.7; 7.2]** |
| **Ureter (R)** | 7.7 [0.1; 78.2] | **7.0 [0.1; 78.8]** | 1.5 [0.0; 34.4] | **0.6 [0.0; 9.3]** |
| **Ureter (L)** | 9.7 [0.1; 79.4] | **8.6 [0.1; 78.7]** | 2.0 [0.0; 34.2] | **0.6 [0.0; 12.8]** |

**Supplementary tables 4a and 4b: Obtained Doses to OARs in the Two Scenarios - Prostate and Pelvic lymph nodes Treatment**

**4a) Anatomical situation with high-volume bladder**

Data are reported as mean dose [range of the DVH curve], in Gy. Bold values indicate doses obtained with optimization.

| **Structure** | **Scenario 5 (IMRT, no optimization)** | **Scenario 6 (IMRT, with optimization)** |
| --- | --- | --- |
| **Bowel** | 0.8 [0.0; 46.8] | **1.7 [0.09; 47.06]** |
| **Bowel V15 Gy** | 59cc | **60 cc** |
| **Sigmoid** | 14.8 [0.61; 46.4] | **14.6 [0.6; 46.4]** |
| **Rectum** | 37.0 [11; 74.6] | **31.0 [8.3; 75.6]** |
| **Bladder** | 37.1 [3.5; 78] | **28.1 [3.4; 77.8]** |
| **Peri-vascular iliac common fat (R)** | 1.4 [1; 2.1] | **1.3 [0.9; 2.0]** |
| **Peri-vascular iliac common fat (L)** | 1.3 [0.3; 2] | **1.3 [0.9; 1.8]** |
| **Peri-vascular iliac external fat (R)** | 34.4 [1.6; 49.4] | **33.9 [1.5; 47.6]** |
| **Peri-vascular iliac external fat (L)** | 28.4 [1.3; 49.3] | **27.9 [1.2; 47.9]** |
| **Bladder dome** | 20.8 [2.7; 46.8] | **19.8 [2.2; 46.7]** |
| **Ureter (R)** | 0.4 [0.12; 70.0] | **20.8 [0.1; 46.0]** |
| **Ureter (L)** | 12 [0.12; 73.8] | **11.0 [0.1; 46.7]** |

**4b) Anatomical situation with low-volume bladder**

Data are reported as mean dose [range of the DVH curve], in Gy. Bold values indicate doses obtained with optimization.

| **Structure** | **Scenario 5 (IMRT, no optimization)** | **Scenario 6 (IMRT, with optimization)** |
| --- | --- | --- |
| **Bowel** | 11.8 [0.2; 67.8] | **12.6 [0.1; 72.7]** |
| **Bowel V15 Gy** | 1100 cc | **1200 cc** |
| **Sigmoid** | 35.7 [16.0; 67.8] | **37.6 [9.9; 72.3]** |
| **Rectum** | 43.0 [12.3; 75.0] | **44.3 [13.3; 77.8]** |
| **Bladder** | 39.3 [14.2; 77.6] | **37.2 [8.7; 79.2]** |
| **Peri-vascular iliac common fat (R)** | 46.2 [44.0; 48.4] | **44.9 [42.5; 47.5]** |
| **Peri-vascular iliac common fat (L)** | 43.5 [43.6; 48.9] | **45.0 [42.4; 48.0]** |
| **Peri-vascular iliac external fat (R)** | 47.5 [44.8; 55.4] | **45.5 [40.8; 52.3]** |
| **Peri-vascular iliac external fat (L)** | 47.6 [44.0; 54.4] | **46.1 [43.7; 52.9]** |
| **Bladder dome** | 32.2 [11.3; 68.2] | **17.8 [8.7; 54.8]** |
| **Ureter (R)** | 36.1 [0.4; 78.5] | **35.0 [0.5; 80.4]** |
| **Ureter (L)** | 36.0 [0.4; 79.2] | **35.1 [0.4; 79.7]** |

**Supplementary text:**

**Technical procedures used for IMRT and SBRT plan preparation:**

**For scenarios refereeing to SBRT using the Cyberknife© system plans:**

Patients were scanned on a GE Discovery RT CT scanner using the acquisition parameters recommended by Accuray™ (120 kV, mAs > 400, 1.25‑mm slice thickness, and a 650‑mm field of view). Patients were positioned supine with their arms placed on the chest. Fiducial markers were implanted in the prostate prior to simulation to enable intrafraction tracking during treatment.

Treatment was delivered using an Accuray™ CyberKnife M6 system equipped with the InCise™ multileaf collimator (MLC), operating at 6 MV (FFF) with a dose rate of 800 MU/min. Treatment plans were generated in the Accuray Precision® Treatment Planning System (TPS) using the VOLO optimizer. Dose calculation was performed with the Full‑Scale Pencil Beam algorithm with lateral scaling.

For beam delivery, a prostate‑optimized template path set was employed to reduce treatment time and promote a well‑distributed beam arrangement around the target volume. Beam path selection was further constrained by the requirement to avoid entry through the external genital organs (EGO). Treatment plans were optimized to meet target coverage and conformity objectives while respecting dose constraints to organs at risk (OARs). Optimization was pushed beyond simple OAR sparing to maximize conformity and dose gradient while maintaining adequate target coverage. Dose to healthy tissues (excluding contoured OARs) was minimized, and a steep dose fall‑off around the target volume was promoted through the use of the Normal Tissue Objective (NTO) or appropriate shell structures.

A treatment plan was considered acceptable when at least 98.0% of the PTV was covered by the prescription isodose (down to 95% when necessary to meet OAR constraints), the CTV received 100.0% coverage, and the conformity index (nCI) was ≤ 1.01, with a total treatment time not exceeding 30 minutes. All target‑volume constraints were required to be met.

**For scenarios referring to IMRT/VMAT plans:**

Patients were scanned on the same CT scanner using the following parameters: 120 kV, 300 mAs, 2.5‑mm slice thickness, and a 500‑mm field of view. Patients were positioned supine with their arms placed on the chest.

Treatment was delivered using a Varian TrueBeam™ linear accelerator equipped with the HD120 MLC. Treatment plans were optimized in the Varian Eclipse™ TPS using the Acuros XB dose calculation algorithm.

Beam delivery consisted of two full arcs (360° rotation) using 10 MV photons with a maximum dose rate of 600 MU/min. The maximum field size along the MLC X‑axis was limited to 15 cm to ensure full leaf interdigitation.

Treatment plans were optimized to achieve target‑volume coverage objectives and meet OAR constraints while minimizing dose to surrounding healthy tissues using the NTO.

A plan was considered acceptable if at least 98% of the PTV received 95% of the prescribed dose and if the maximum dose to 2% of the PTV remained below 105% of the prescription dose.
